# Effects of Renal Sympathetic Denervation on Post-Myocardial Infarction Cardiac Remodeling in Rats

**DOI:** 10.1371/journal.pone.0045986

**Published:** 2012-09-26

**Authors:** Jialu Hu, Meng Ji, Conway Niu, Asiyeguli Aini, Qina Zhou, Ling Zhang, Tao Jiang, Yan Yan, Yuemei Hou

**Affiliations:** 1 Department of Cardiology, Zhongshan Hospital, Fudan University, Shanghai, China; 2 Department of Cardiology, the First Affiliated Hospital of Xinjiang Medical University, Xinjiang, China; 3 Shanghai Medical College, Fudan University, Shanghai, China; 4 Department of Cardiology, the Central Hospital of Fengxian District, Shanghai, China; Temple University, United States of America

## Abstract

**Objective:**

To investigate the therapeutic effects of renal denervation (RD) on post- myocardial infarction (MI) cardiac remodeling in rats, the most optimal time for intervention and the sustainability of these effects.

**Methods:**

One hundred SPF male Wistar rats were randomly assigned to N group (Normal, n = 10), MI group(MI, n = 20),RD group (RD, n = 10), RD3+MI (MI three days after RD, n = 20), MI1+RD (RD one day after MI, n = 20), MI7+RD (RD seven days after MI, n = 20). MI was produced through thoracotomic ligation of the anterior descending artery. RD was performed through laparotomic stripping of the renal arteriovenous adventitial sympathetic nerve. Left ventricular function, hemodynamics, plasma BNP, urine volume, urine sodium excretion and other indicators were measured four weeks after MI.

**Results:**

(1) The left ventricular function of the MI group significantly declined (EF<40%), plasma BNP was elevated, urine output was significantly reduced, and 24-hour urine sodium excretion was significantly reduced. (2) Denervation can be achieved by surgically stripping the arteriovenous adventitia, approximately 3 mm from the abdominal aorta. (3) In rats with RD3+MI, MI1+RD and MI7+RD, compared with MI rats respectively, the LVEF was significantly improved (75±8.4%,69±3.8%,73±5.5%), hemodynamic indicators were significantly improved, plasma BNP was significantly decreased, and the urine output was significantly increased (21.3±5 ml,23.8±5.4 ml,25.2±8.7 ml). However, the urinary sodium excretion also increased but without significant difference.

**Conclusions:**

RD has preventive and therapeutic effects on post-MI cardiac remodeling.These effects can be sustained for at least four weeks, but there were no significant differences between denervation procedures performed at different times in the course of illness. Cardiac function, hemodynamics, urine volume and urine sodium excretion in normal rats were not affected by RD.

## Introduction

Acute coronary occlusion results in the loss of myocardial tissue, the inhibition of myocardial function, and low blood pressure. This leads to the activation of a series of neurohormonal factors, such as the baroreceptor-mediated sympathetic nervous system, the renin-angiotensin-aldosterone system (RAAS), endothelin, and tumor necrosis factor, which increases the heart rate, myocardial contractility, vasoconstriction, and fluid retention to help maintain hemodynamic stability. However, in this case, these intended mechanisms for short-term maintenance of blood pressure caused sustained activation, and will lead to left ventricular remodeling and deterioration of left ventricular function. Of these mechanisms, the chronic activation of the sympathetic nervous system is the main mechanism of post-MI heart failure. [1], [2].

In recent years, some clinical trials have demonstrated that RD has improved sodium and water retention and the chronic over-activation of the sympathetic nervous system, achieving a breakthrough in the treatment of refractory hypertension. [3], [4]Furthermore, increasing evidence has suggested that blockade of the sympathetic nervous system and RAAS have clear results on effects related to heart failure. [5], [6], [7] This suggests to us that RD has great potential in the treatment of heart failure, cardiorenal syndrome, cirrhosis, insulin resistance, and a series of illnesses related to chronic sympathetic activation or sodium and water retention.

In 2002, Nozawa et al. explored the role of RD in heart failure, and their study proved that RD improves left ventricular remodeling and function, and increases water and sodium excretion. [8] To our knowledge, no other authors except Nozawa et al., at present, have reported similar results, probably due to lack of a clinically feasible RD approach, limiting the value of research. We hope better animal research would confirm the efficacy of RD intervention in MI-induced cardiac remodeling, and we want to know the optimal time for performing RD and the sustainability of these effects.

In the present study, we used six groups of rats: RD3+MI, MI1+RD, MI7+RD, MI, RD, and Normal. After four weeks of observation, body weight, left ventricular weight, left ventricular function, hemodynamics, plasma BNP, urine volume, urine sodium, and other indicators were measured. By comparing the use of RD treatment at different times in the course of illness, we were able to find the most optimal time to use RD in post-MI cardiac remodeling and confirm experimental evidence of the sustainability of RD.

## Methods

### Ethics Statement

The experiment scheme and the use of rats were approved by animal use and management ethics committee of the First Affiliated Hospital of Xinjiang Medical University (batch: IACUC-20110325017). Experimental design and the implementation process were undertaken in accordance with animal welfare guidelines, 3 R principles, and AAALAC (Association for Assessment and Accreditation of Laboratory Animal Care) standards.

### Animal

One hundred male Wistar rats (SPF level, 280–330 g) were obtained from the Experimental Animal Center of Xinjiang Medical University. These rats were housed in groups at a temperature of 22–25°C in a 50 to 70% humidity controlled room with a 12-hour light/dark cycle. A standard rat diet containing 0.3% NaCl and tap water were given ad libitum throughout the experimental period.

### Groups Setting

The rats were randomly assigned to six groups (rats were numbered in descending order, complete randomization method was used and a random number was produced by the CHISS statistical software), i.e., MI group (simple MI) n = 20, RD group (renal denervation) n = 10, N group (Normal) n = 10, RD3+MI (MI three days after renal denervation) n = 20, MI1+RD (renal denervation one day after MI) n = 20, MI7+RD (renal denervation seven days after MI) n = 20.

### Induction of Myocardial Infarction

MI was induced by ligation of the left coronary artery under combined intraperitoneal anesthesia (Ketamine (75 mg/kg), diazepam (5 mg/kg), atropine (0.05 mg/kg)). Endotracheal intubation was followed by mechanical ventilation. A left thoracotomy was performed to exteriorize the heart. The left anterior descending coronary artery was ligated together with a small amount of myocardial tissue approximately 2–3 mm from its origin with a 7–0 silk suture. Hallmarks of success for the white surface of the heart, the heart beat decreased and ECG T-wave changes occurred. Intraperitoneal injection of 1% lidocaine (1 mg/kg) was given if ventricular fibrillation occurred. The thorax was closed, and after cardiopulmonary resuscitation and restoration of spontaneous breathing, rats were placed in 28°C environment. Half an hour later ECG (I, II, III, avF, avL, avR, V1-lead) was retested; ST segment elevation was recognized as a successful model. After rats were awoken, they were given daily pethidine 50 mg and penicillin 200,000 U intramuscularly for 3 days. Infarct size of >30% was deemed as successful in this model.

### Renal Denervation

Bilateral RD was performed under intraperitoneal anesthesia (Ketamine (75 mg/kg), diazepam (5 mg/kg), atropine (0.05 mg/kg)). Bilateral flank incisions were made, and RD was performed surgically by stripping the adventitia of renal arteries and veins at about 3 mm from the abdominal aorta for 1–2 cm and dissecting all visible renal nerve bundles, and coating the vessels with a solution of 10% phenol in ethanol. We evaluated the effects of RD by electrically stimulating (Grass S48 nerve stimulator, 15 V, 0.2 ms, 10 Hz) the renal sympathetic nerve at the proximal renal artery for 10–30 s before and after renal denervation, respectively, to observe the disappearance of sympathetic effects. In normal rats, electrical stimulation can cause the blood pressure to increase 5–10 mm Hg, the heart rate to increase 8–15 bpm, and the kidney to become paler in color. When electrically stimulated after RD, sympathetic effects were absent with no apparent changes in heart rate, blood pressure, or color of the kidney.

### Measurement of Urine Volume and Sodium Excretion

The rats were moved to individual metabolic cages with free access to tap water. Urine was collected for 24 h. After the measurement of urinary volume, the rats were returned to the original cage. Urine sodium concentration was measured by electrolyte analyzer IMS-972Plus (Xi Cai Heng Medical Electronics, China), using the ion-selective electrode method.

### Detection of Left Ventricular Function

Four weeks after MI induction, transthoracic echocardiography was performed under ether anesthesia with a high-resolution small animal echocardiographic system equipped with a Heart RMV710 probe (frequency 37.5 Hz, axial resolution of 70 u, focal length 15 mm, effective viewing area 10.7 mm). A two-dimensional short-axis view of the left ventricle (LV) was obtained at the level of the papillary muscle and two-dimensional targeted M-mode tracings were recorded. End-diastolic and end-systolic LV internal dimensions and fractional shortening and ejection fraction were determined from at least three consecutive cardiac cycles.

### Hemodynamic Study

After echocardiographic data collection, a 2-F micromanometer-tipped catheter was inserted into the right carotid artery and advanced into the LV to determine LV pressure. With the rat anesthetized lightly and breathing spontaneously, LV pressure was recorded on a multichannel electrophysiology instrument (LEAD-2000, Sichuan Jinjiang Electronic Science and Technology Co., Ltd., China). Heart rate (HR), left ventricular systolic pressure (LVSP), LV end-diastolic pressure(LVEDP), maximum and minimum values of rate of change in LV pressure (dP/dt_Max_ and dP/dt_Min_) were obtained from the signal processing computer system of the multichannel electrophysiology instrument.

### Determination of the Proportion of Plasma BNP, Weight, Left Ventricular Mass Index, and Left Ventricular Infarct Ratio

After the hemodynamic study, weighing of rats and opening of the thorax to obtain blood directly from the heart, plasma BNP content was measured by radioimmunoassay. After collection of blood, the heart was immediately removed and washed in normal saline, dried with filter paper, and weighed using an electronic scale to obtain the left ventricular mass (including interventricular septum). The left ventricular mass index was calculated and the left ventricular infarct area ratio was determined.

### Statistical Analysis

Results were expressed as mean ± SD. Group comparisons were made with analysis of variance (ANOVA), followed by the LSD test to identify differences among various groups. A value of P<0.05 was considered statistically significant.

## Results

### Weight, Left Ventricular Mass Index, and Left Ventricular Infarct Area Ratio after MI

There was no significant difference in weight, left ventricular mass index, and infarct area ratio for each group four weeks post-MI. (See Table 1).

**Table 1:** Body weight, left ventricular mass index, and infarct size four weeks after MI.

	Weight(g)	LV Mass Index (mg/cm^2^)	Infarct size(%)
N	311±46	32.1±2.3	
RD	314±49	33.5±1.1	
MI	318±58	34.1±2.3	36.0±6.2
RD3+MI	306±40	34.2±2.5	35.2±7.4
MI1+RD	302±59	35.3±2.6	38.1±5.3
MI7+RD	336±73	36.2±3.4	39.0±8.1

Data are means ± SD obtained four weeks post-MI. *P<0.05 vs N, #P<0.05 vs MI.

### The Effect of RD on Cardiac Function

Four weeks post-MI, EF and FS of MI group were significantly reduced (87±4.2 and 42±5.6 versus 37±6% and 16±3%, P<0.05). EDV and ESV were significantly increased(0.72±0.19 ml and 0.1±0.04 ml versus 2.16±0.68 ml and 1.37±0.042 ml, P<0.05). EF and FS in RD3+MI group, MI1+RD group and MI7+RD group, compared with MI group, were significantly increased, but were lower than normal (P<0.05). EDV and ESV of these three groups were found to be significantly lower than the MI group (P<0.05). (See Table 2).

**Table 2:** Impact on cardiac function.

	LVEF (%)	LVFS (%)	LVEDV (ml)	LVESV (ml)
N	87±4.2	42±5.6	0.72±0.19	0.10±0.04
RD	79±4.8 #	39±3.5 #	0.77±0.26 #	0.18±0.11 #
MI	37±6.0 *	16±3.0 *	2.16±0.68 *	1.37±0.04 *
RD3+MI	75±8.4 *#	39±7.5 *#	0.72±0.19 #	0.19±0.10 *#
MI1+RD	69±3.8 *#	34±2.8 *#	0.81±0.17 #	0.22±0.07 *#
MI7+RD	73±5.5 *#	38±4.4 *#	0.75±0.19 #	0.20±0.08 *#

Data are means ± SD obtained four weeks post-MI. *P<0.05 vs N, #P<0.05 vs MI.

### The Effect of RD on Hemodynamics

We observed that rats in the RD intervention groups, compared with rats in the simple MI group, had increased LVSP (P<0.05), decreased LVEDP (P<0.05), and increased dP/dt_Max_ (P<0.05) and dP/dt_Min_ (P<0.05). Improvements in RD3+MI and MI1+RD groups were better than the MI7+RD group, but did not reach statistical significance (P>0.05). (See Table 3).

**Table 3:** Impact on Hemodynamics.

	HR(bpm)	LVSP(mmHg)	LVEDP(mmHg)	dP/dt _Max_(×10^3^ mmHg/s)	dP/dt _Min_(×10^3^ mmHg/s)
N	455.64±18.76	120±8	3±3	10.8±1.1	6.2±0.8
RD	459.72±16.76	122±7	4±2	11.0±0.9	6.3±0.6
MI	460.89±13.53	95±5*	24±6*	5.2±0.6*	3.2±0.4*
RD3+MI	451.12±14.23	114±10#	10±4*#	8.8±1.0#	5.1±0.5#
MI1+RD	453.00±16.66	116±7#	11±4*#	9.2±1.4#	5.3±0.7#
MI7+RD	456.45±15.52	111±9#	14±3*#	8.0±0.4*#	4.9±0.4*#

Data are means±SD obtained four weeks post-MI. *P<0.05 vs N, #P<0.05 vs MI.

### The Effect of RD on Urine Volume and Urine Sodium

**Figure 1:**
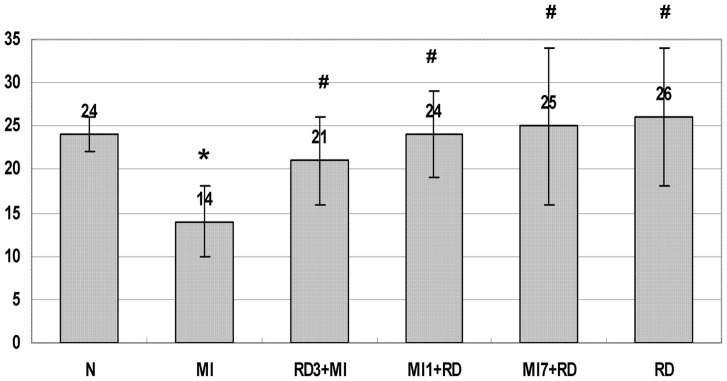
The effect of RD on urine volume (ml/day). Data are means ± SD obtained four weeks post-MI. *P<0.05 vs N, #P<0.05 vs MI.

**Figure 2:**
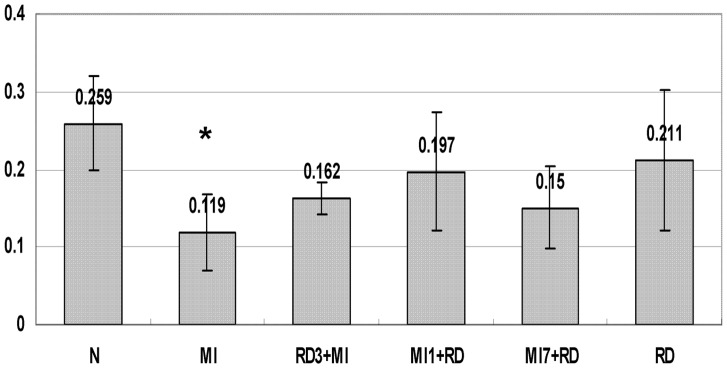
The effect of RD on urine sodium excretion (mmol/day). Data are means ± SD obtained four weeks post-MI. * P<0.05 vs N.

Four weeks post-MI, compared with the normal control group, urine output in the MI group was significantly reduced from 23.83±1.9 ml to 14.13±3.8 ml and urinary sodium excretion was decreased from 0.259±0.061 mmol to 0.138±0.019 mmol (P<0.05). As shown in Figure 1, RD rats without MI did not have changes in urine output or urinary sodium excretion. Compared with the MI group respectively, RD had improved the impaired urine output significantly while excretion of urinary sodium did not differ in RD3+MI group, MI1+RD group, and MI7+RD group (See Figure 2).

### The Effect of RD on Changes in Plasma BNP

**Figure 3:**
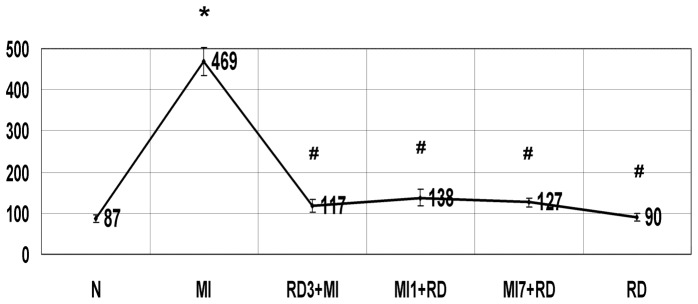
The effect of RD on changes in plasma BNP (pg/ml). Data are means ± SD obtained four weeks post-MI. *P<0.05 vs N, #P<0.05 vs MI.

When comparing MI group rats four weeks post-MI with N group, there is a significant increase, but there was no significant difference between the RD group and N group.The RD3+MI, MI2+RD, and MI7+RD groups had significantly decreases when compared with the MI group respectively, but were all higher than the N group (P<0.05), with no significantly differences amongst the groups (See Figure 3).

## Discussion

The present study demonstrated that RD, whether performed before or after MI, can both prevent and lessen the deterioration of LV function and LV dilatation post-MI, including significantly increased EF, LVSP, dP/dt_Max_, and dP/dt_Min_, while LVEDV, LVESV, and LVEDP decreased significantly compared with the MI group, as shown in Tables 2 and 3.

RD before MI may reduce excessive sympathetic response in the acute phase of MI. Clinical studies have demonstrated that β-adrenergic receptor blockade is beneficial in heart failure due to the decreased cardiac sympathetic stress that could reduce myocardial oxygen consumption, myocardial damage, and myocardial cell apoptosis. [9]–[11].

The process of ventricular enlargement can be influenced by infarct size, ventricular wall stress, and neurohumoral factors, including adrenergic drive and the renin-angiotensin system. [12], [13] According to previous studies, an increase in sympathetic activity after MI might contribute to the progression of heart failure [14]; cardiac adrenergic activity may be less in MI rats with RD than in rats with MI, because of lower LV end-diastolic pressure in RD rats [8]; In addition, angiotensin-converting enzyme inhibition, which might reduce both volume and pressure overload, attenuates ventricular remodeling post-MI. [15] Considering all the above findings, in the present study, lower LVEDP and smaller LVEDV in MI rats with RD due to the restoration of natriuresis or the blocking of sympathetic system and RAAS by RD may be an important component in the prevention of ventricular remodeling and improvement of LV function post-MI.

There was a significant decrease in BNP levels in MI rats with RD compared with MI group four weeks post-MI, with no significant differences among the RD groups. This result again confirms that RD actually reduced the LV filling pressure and lessened post-MI cardiac remodeling.

MI rats had severe water and sodium retention at four weeks post-MI. Urine volume and urinary sodium excretion significantly reduced. The results are consistent with previous animal studies and the established pathogenic mechanism of chronic heart failure. [16] Retention of fluid and sodium is one of the most important characteristics of heart failure, caused by the joint action of a variety of capacity-control mechanisms including the renin-angiotensin-aldosterone system, prostaglandins, natriuretic peptides, vasopressin and sympathetic nervous system. [17] Among all these mechanisms, activation of the sympathetic nervous system is the most important one. As renal sympathetic output increases, renal blood flow is reduced and sodium and water reabsorption is enhanced; in addition, sympathetic activation can increase renin secretion and the resistance of the renal efferent artery. [18] Studies have demonstrated that water and sodium retention in heart failure can be amplified by the activation of the sympathetic system which leads to the disappearance of urinary sodium secretion. [19], [20] Our results confirm that RD improves water secretion significantly, however, the effect on urinary sodium excretion failed to reach significant differences. Interestingly, contrary to our findings, Nozawa et al. found that RD could result in a significant increase in urinary sodium secretion in rats with heart failure, but had no significant impact on urine output**.** [8] In regards to this difference, we concluded that the effect of RD on urine output and sodium excretion is the same in both experiments, with the only difference in its extent. The reason behind this may be in the process of collecting the urine and the sample size.

Finaly, when compared with the normal group, rats of the RD group had slight increases in HR, and slight decreases in heart function and 24-hour urine sodium. We are considering that this may be due to the side effects of the RD procedure itself (such as the trauma caused by the procedure) and variation among the animal subjects, causing a greater standard deviation. Because there was no significant statistical difference, we believe that RD does not have a significant effect on normal rats.

### Limitations

We know that myocardial infarction expansion, left ventricular dilation, and compensatory myocardial hypertrophy in non-infarcted regions are involved in ventricular remodeling after coronary occlusion. In our study, the MI group underwent significant cardiac function and hemodynamic changes compared with normal group. However, changes were not statistically significant when compared with the indicator of LVMI. Our findings were similar to those of Byung-Hee [21] and Pfeffer [22]. The hypertrophy of myocardial fibers in non-infarcted and border zones were found in rats after two hours of coronary occlusion, but there was a lack of significant LVMI change between sham and MI groups. [21] In moderate and large infarcts as inflammation and edema developed, LV weight increased, then progressively decreased as a thin scar formed, then returning to normal values as a result of compensatory hypertrophy of the residual myocardium. [22] In Nowaza’s study, the findings were also similar: there was no significant change in LVMI one month post-MI. [8].

It is our deduction that this phenomenon may be due to a few factors. Primarily, different infarct sizes and different timing of measurements may explain some variation in results, since heart weight, especially the LV weight, depends on the balance between tissue loss in the infarcted region and compensatory hypertrophy of surviving myocardium. This indicates that if the changes in LVMI during remodeling are to be observed, other non-infarction heart failure models may be necessary.

In addition, there were also limitations to standardization by weight, since weight might be affected by water and sodium retention. When collecting samples, there were variations in the amount of interventricular septal tissue collected, and there were also variations in degree of drying after washing with normal saline. Another factor may have been insufficient observation time.

Due to lack of detection techniques and experimental scales, we did not measure indices such as blood or tissue norepinephrine or β receptors, which reflected the activity of the sympathetic nervous system and the reconstruction of the sympathetic nervous system. Blood content changes of RAAS components were not measured either. Besides BNP, indices such as endothelin were not able to be measured. These indices are very important to RD research in post-MI heart failure.

Therefore, after it was confirmed that RD improves left ventricular remodelingcardiac function, water and sodium excretion in rats with post-MI heart failure, an in-depth study should be conducted in further studies.

### Conclusions

Our study demonstrates that RD has a clear preventive and therapeutic effect on post-MI cardiac remodeling through lessening left ventricular remodeling, increasing cardiac function and water excretion, decreasing BNP levels, with the effect lasting at least four weeks. There were no significant differences for denervation procedures that took place at different times in the course of illness. Cardiac function, hemodynamics, BNP levels, urine volume and urine sodium discharge in normal rats were not affected by RD.

### Clinical Implications

The primary mechanisms of heart failure after AMI include ventricular remodeling and cardiac sympathetic remodeling, chronic over-activation of the sympathetic nervous system, renal sympathetic nerve activation and secondary RAAS activation. [23] Based on previous studies, it was hypothesized that RD could prevent chronic renal afferent and efferent sympathetic activation in heart failure, thus blocking central sympathetic activation and reducing overall activity of the sympathetic nervous system. In addition, the activation of RAAS was also prevented through the inhibition of renin release and blocking the effect of angiotensin II secondary to the activation of RAAS that causes chronic activation of the sympathetic nervous system in the whole body. [24] Thus inhibiting chronic activation of the sympathetic nervous system by blocking local sympathetic nerves such as renal sympathetic nerves, leads to RD having the same effect as sympathetic blockade medications on improving cardiac sympathetic system reconfiguration, reducing myocardial oxygen consumption and ischemic injury, and reducing blood pressure to improve the heart preload and afterload. However, unlike sympathetic blockade medications, RD could avoid a number of adverse reactions such as decreased cardiac contractility. Clinical studies suggest that β-blockers could prolong survival time of heart failure patients with post-MI renal insufficiency. [25] However, patients with severe renal insufficiency will have to avoid using the β-blockers that have to be excreted by the kidneys. [26] This highlights the importance of RD as a new non-drug treatment that blocks the sympathetic nervous system in heart failure.

However, previous basic and clinical studies indicate that even though surgical renal denervation causes great trauma, and that there are many complications or adverse effects related to the procedure itself or neurovascular damage, the effects of RD are complete and precise. [27], [28] Many clinical studies have confirmed that RD via percutaneous radiofrequency catheter ablation is a minimally invasive procedure and an effective RD method, with few complications or adverse effects after long-term observation. [3], [4] However, the problem is that there is difficulty ensuring full effective ablation, and previous animal studies have shown neural regeneration. [28] If percutaneous catheters do not completely ablate the nerve, it could shorten the effective duration of RD. Further understanding of RD by radiofrequency ablation is needed.
